# Why people failed to adhere to COVID-19 preventive behaviors? Perspectives from an integrated behavior change model

**DOI:** 10.1017/ice.2020.245

**Published:** 2020-05-15

**Authors:** Derwin K. C. Chan, Chun-Qing Zhang, Karin Weman-Josefsson

**Affiliations:** 1 Faculty of Education and Human Development, The Education University of Hong Kong, Hong Kong SAR, China; 2 School of Psychology, Curtin University, Perth, Australia; 3 School of Public Health, The University of Hong Kong, Hong Kong SAR, China; 4 Department of Sport and Physical Education, Hong Kong Baptist University, Hong Kong SAR, China; 5 Center for Research on Welfare, Health and Sport, Halmstad University, Halmstad, Sweden

*To the Editor*—Many preventive behaviors such as the practice of hand, personal, and respiratory hygiene; maintaining social distance (eg, staying home); and cleaning and disinfection are recommended for the prevention of the new coronavirus (COVID-19). However, a growing number of reports have revealed individuals’ violations to these COVID-19 preventive behaviors.^1^ These violations might endanger the community by increasing the risk of an outbreak of COVID-19. The uptake of and adherence to health behaviors, including behaviors related to the prevention of infectious diseases (eg, COVID-19), are likely highly dependent on individuals’ motivation, intention, and other decision-making factors.^2^ We aim to apply an integrated behavior change model of health psychology to explain why individuals fail to comply and adhere to these behaviors.

## The integrated model

**Fig. 1.**
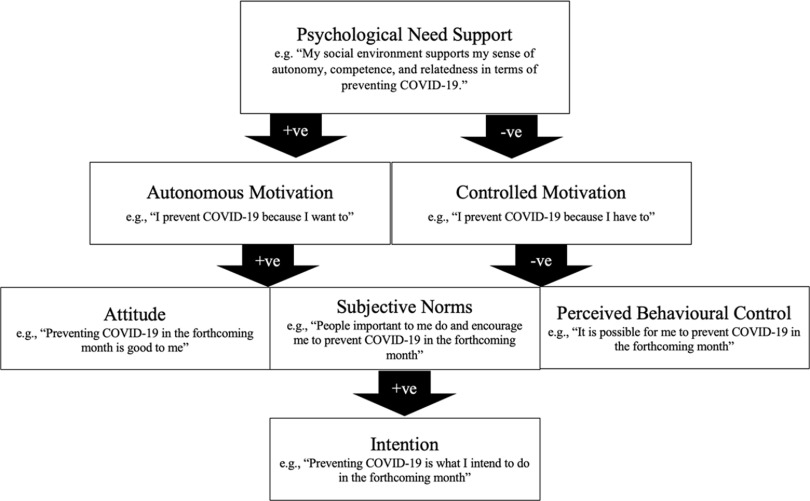
The integrated model of self-determination theory and the theory of planned behavior.

The integrated model of self-determination theory^3^ and the theory of planned behavior^4^ is a behavior change model that utilizes the concepts of 2 widely used psychological theories. The integrated model outlines the processes by which psychological need support, and motivations directly and indirectly link to the social cognition beliefs, intention, and behavior (Fig. 1).^5^ In the integrated model,^5^ when social environments are supportive to individuals’ basic psychological needs of autonomy, competence, and relatedness, individuals are more likely to endorse autonomous motivation (ie, acting for inherent interest, satisfaction, personal goals, and values) than controlled motivation (ie, acting due to external contingencies, internal pressure, or sense of ego). They are also more likely to have more favorable social cognition beliefs (ie, attitude, subjective norm, and perceived behavioral control) and intentions and to demonstrate behavioral adherence in health behaviors. The psychological pathways illustrated in the integrated model have been supported by evidence from various health contexts and cultures,^5,6^ including preventing H1N1 transmission during a pandemic.^2^ We believe that the integrated model can explain why some people have failed to adhere to the recommended behaviors for COVID-19 prevention.

## Why people do or do not adhere to COVID-19 prevention recommendations

### Law enforcement

A number of countries have set up legislation regarding social distance measures (eg, stay-home restriction), quarantine, and lockdown/travel ban. These legislative actions are classic examples of external factors that foster the development of controlled motivation. According to the integrated model,^5^ individuals who are driven by controlled motivation (ie, acting due to external contingencies, internal pressure, or sense of ego) may adhere to the advisory behavior as soon as the external factors (eg, contingencies of following COVID-19 preventive behaviors or not) are present, but they are more vulnerable to nonadherence in the long term than those who hold autonomous motivation (ie, acting for inherent interest, satisfaction, personal goals, and values) for the action. Individuals driven by controlled motivation alone might consider violations of health legislations when they perceive that the risks of getting caught or negative health consequences are low.^7^ At present, the enforcement of some new COVID-19 prevention legislations (eg, social distancing measures) could be extremely challenging when the surveillance involves a large geographical area or population. Governments or public health organizations should consider noncoercive strategies that are aligned with basic psychological needs to foster individuals’ autonomous motivation of COVID-19 prevention.

### Social and environmental factors

In addition to law enforcement, other social situations and environmental factors are supportive or detrimental to the motivational and social cognition factors affecting COVID-19 prevention. In support to the psychological factors in the integrated model^5^, there are social situations or personal beliefs that facilitate autonomous motivation (eg, “preventing COVID-19 is what I want to do because I am responsible for my own health”), attitude (eg, accessible online information about the values of COVID-19 prevention), subjective norms (eg, family or friends who are following the COVID-19 preventive strategies say I should do the same), and perceived behavioral control (eg, training resources that make it easier for me to correctly apply COVID-19 preventive behavior such as hand hygiene). In contrast, some social circumstances are detrimental to motivational and social cognition factors. For instance, advice on the necessity of wearing face masks in community settings has been inconsistent across different nations and health organizations,^8^ which might discourage individual autonomous motivations (eg, “Do I really want to prevent COVID-19 in this way?”) and attitudes (eg, “Are there any points to wearing a face mask for the prevention of COVID-19?”). The shortage of personal protective equipment (PPE)^9^ might impair an individual’s sense of competence and perceived behavioral control (eg, “lack of PPE has made the prevention of COVID-19 challenging and uncontrollable”). Discrimination toward, alienation of, and labeling of individuals who wear face masks in public areas^10^ or social groups that encourage the ignorance of social distance measures^1^ might undermine an individual’s relatedness and subjective norms in the context of COVID-19 prevention. Governments and health organizations should be aware of these factors and should implement policies and social strategies that facilitate the motivational and social cognition factors affecting COVID-19 prevention.

In conclusion, the integrated model of self-determination theory and the theory of planned behavior explains why some individuals fail to adhere to the preventive behaviors of COVID-19. We hope our discussion may raise the awareness of governing bodies and public health sectors regarding the importance of considering individuals’ motivation and social cognition beliefs when implementing COVID-19 preventivon measures in the community.
